# Insights Into Peptide Inhibition of Alpha-Synuclein Aggregation

**DOI:** 10.3389/fnins.2020.561462

**Published:** 2020-10-15

**Authors:** James H. Torpey, Richard M. Meade, Ravina Mistry, Jody M. Mason, Jillian Madine

**Affiliations:** ^1^Institute of Integrative Biology, University of Liverpool, Liverpool, United Kingdom; ^2^Department of Biology and Biochemistry, University of Bath, Bath, United Kingdom

**Keywords:** alpha-synuclein, electron microscopy, neurodegenerative disease, NMR, Parkinson disease, peptide interaction, protein aggregation

## Abstract

α-Synuclein (aSyn) aggregation is an attractive target for therapeutic development for a range of neurodegenerative conditions, collectively termed synucleinopathies. Here, we probe the mechanism of action of a peptide 4554W, (KDGIVNGVKA), previously identified through intracellular library screening, to prevent aSyn aggregation and associated toxicity. We utilize NMR to probe association and identify that 4554W associates with a “partially aggregated” form of aSyn, with enhanced association occurring over time. We also report the ability of 4554W to undergo modification through deamidation of the central asparagine residue, occurring on the same timescale as aSyn aggregation *in vitro*, with peptide modification enhancing its association with aSyn. Additionally, we report that 4554W can act to reduce fibril formation of five Parkinson’s disease associated aSyn mutants. Inhibitory peptide binding to partially aggregated forms of aSyn, as identified here, is particularly attractive from a therapeutic perspective, as it would eliminate the need to administer the therapy at pre-aggregation stages, which are difficult to diagnose. Taken together the data suggest that 4554W could be a suitable candidate for future therapeutic development against wild-type, and most mutant aSyn aggregation.

## Introduction

Neurodegenerative diseases, such as Parkinson’s disease (PD), are associated with the self-assembly and aggregation of proteins (Goedert, 1999). They arguably represent one of the most significant challenges to modern medicine; they are prevalent and, as yet, there are no tools available to fight back against the gradual yet relentless progression of neurodegeneration. A range of strategies targeting toxicity associated with protein aggregation are being investigated (Dehay et al., 2015; Fields et al., 2019). These include the use of passive or active immunization (Lee and Lee, 2016; Vaikath et al., 2019). Another strategy is reduction of protein expression or enhancement of clearance mechanisms (Stefanis et al., 2019), together with methods to prevent cell to cell transmission of misfolded proteins (Hasegawa et al., 2017). Targeting the aggregation process directly presents an attractive therapeutic option and has been probed using chaperones (Arosio et al., 2016; Burmann et al., 2020) or via modulation and inhibitor compounds (Singh et al., 2017; Pujols et al., 2018; Peña-Díaz et al., 2019). The majority of drugs currently on the market are small molecules, however there is an increasing level of interest in the use of peptides as pharmaceuticals. Peptides are generally more specific to a given target than small molecules and thus are considered less likely to have prohibitive side-effects. Many of the issues that have historically proved an impediment to the clinical development of peptides (e.g., proteolytic degradation and localization) are better understood and can now be circumvented (Mason, 2010; Fosgerau and Hoffmann, 2015). Peptides are particularly attractive to neurodegenerative research associated with protein aggregation due to their potential to impede these broad and shallow protein-protein interactions, and therefore fibril formation. Some peptides have been shown to form β-hairpin structures that can “cap” fibrils to prevent the addition of further monomeric units and thus blocking their elongation, as shown for amylin and α-synuclein (aSyn) (Huggins et al., 2011). An alternative approach has been to inhibit assembly on one side of the protein using N-methylated peptides to prevent the formation of hydrogen bonds (Madine et al., 2008) or using short peptides containing residues that prevent further protein attachment (Kim et al., 2009). There is therefore great interest in the development of new peptide-based inhibitors to combat the toxicity of aggregation-prone proteins, such as aSyn (Mason and Fairlie, 2015).

A 10-residue peptide candidate, 4554W (KDGIVNGVKA) has been previously identified by an intracellular library-screening technique, and showed a reduction in aSyn fibril formation and reduced cytotoxicity (Cheruvara et al., 2015). However, this work did not provide insight into the mode of action of the peptide. The work presented here aimed to employ NMR techniques to test the hypothesis that 4554W bound to aSyn, and to identify key residues involved in the interaction. The ability of 4554W to inhibit the fibrillation of PD-associated aSyn mutants was also explored. It was found that 4554W associates with partially aggregated forms of aSyn, preventing fibril growth. This is appealing for further therapeutic development since the mechanism need not inhibit the native function of aSyn, and could be administered following identification of disease initiation, instead of at a pre-diagnostic stage when no aggregation has occurred.

## Materials and Methods

### Expression of aSyn

The pRK172 aSyn expression construct (kindly gifted by Michel Goedert) was freshly transformed into *E. coli* BL21 (DE3) cells, using the heat-shock method. These cells were used to inoculate 1 mL of super optimal broth with catabolite repression (SOC) (100 μg/mL ampicillin), which was grown at 37°C with shaking at 200 rpm for 8 h. 150 μL of this culture was used to inoculate 50 mL of minimal medium (Solution A: 12.5 g/L Na_2_HPO_4_, 7.5 g/L KH_2_PO_4_ pH 7.2; Solution B (for 1 L): 4 g glucose, 1 g (^15^N) NH_4_Cl, 240 mg MgSO_4_⋅7H_2_O, 20 mg CaCl2⋅2H_2_O, 10 mg thiamine), and grown at 37°C overnight. This starter culture was used to inoculate 1 L of minimal medium such that the starting optical density at 600 nm (OD600) was 0.1, with growth at 37°C with shaking at 180 rpm until the OD600 reached 0.8. At this point isopropyl-β-D-1-thiogalactopyranoside (IPTG) was added to the culture to a final concentration of 0.5 mM and the culture was then incubated with shaking overnight at 18°C. The cells were harvested by centrifugation at 4,000 g for 20 min at 4°C. The cell pellets were snap frozen in liquid nitrogen (LN_2_) prior to storage at −80°C.

### Site-Directed Mutagenesis of aSyn

The QuikChange II kit (Agilent Technologies) was used according to the manufacturer’s instructions to prepare the six PD-linked aSyn mutants (A30P, E46K, H50Q, G51D, A53T, and A53E). The WT aSyn pRK172 expression construct was used as the template and the reaction carried out according to the manufacturer’s instructions with the primer sequences shown in Supplementary Table 1. Successful mutagenesis was confirmed by sequencing (Source Bioscience) and proteins expressed and purified as for wild-type.

### Purification of aSyn

Cell pellets were resuspended in 20 mL Buffer A [20 mM Tris-HCl pH 8.0, 1 mM ethylenediaminetetraacetic acid (EDTA)], and lysed by pressure homogenization, followed by a single cycle of ultra-sonication (30 s at 23 kHz). The lysate was incubated at 85°C for 10 min and then clarified by centrifugation at 18,000 g for 30 min at 4°C. The clarified lysate was applied directly to a 5 mL Q HiTrap anion exchange chromatography column (GE Healthcare Life Sciences) pre-equilibrated with Buffer A. Protein was eluted from the column via gradient elution with Buffer B (Buffer A + 1M NaCl). aSyn elutes from the column at approximately 300 mM NaCl. Fractions were analyzed by SDS-PAGE, pooled and filtered through an Amicon Ultra-15 centrifugal filter with a 30 kDa molecular weight cut-off (MWCO) (EMD Millipore). The flow-through was collected and applied to a 10 kDa MWCO centrifugal filter, and concentrated to 10 mg/mL. Protein concentration was determined using UV absorbance at 280 nm and purity assessed by SDS-PAGE and mass spectrometry. aSyn was buffer exchanged into double-distilled water (ddH_2_O) using a PD-10 desalting column (GE Healthcare Life Sciences) and lyophilized. Lyophilized protein was monomerised by resuspension in hexafluoroisopropanol (HFIP) and thoroughly vortexed until transparent. The HFIP was then evaporated under a stream of nitrogen and resuspended in the required buffer.

### Production and Purification of Peptides

4554W was synthesized using a Liberty Blue microwave peptide synthesizer (CEM). The peptide was synthesized on a Rink amide ChemMatrix resin (PCAS BioMatrix) employing Fmoc solid-phase technique, with repeated steps of coupling-deprotection-washing for each amino acid. The activator solution consisted of 26 g PyBOP in 100 ml DMF, and the deprotection solution was 20% Piperidine in DMF with the addition of 5% Formic acid to prevent aspartamide formation of the peptide.

The peptide was removed from the matrix by incubating in cleavage solution (95% TFA, 2.5% Triisopropylsilane, and 2.5% water), on a shaker at 25°C, for 4 h. The resin was removed by filtration, and the peptide precipitated using ice cold ether, with vortexing and centrifugation at 7,000 g for 3 rounds. The pellet was left overnight at room temperature to completely dry, and purified by HPLC using a Jupiter 4 μm Proteo C-18 90 Å reverse phase semipreparative column.

The fractions of the HPLC peaks were examined by mass spectroscopy, using a microTOF (Bruker Daltonics) to confirm which fractions contained the purified peptide (Supplementary Figure 1). Fractions, containing the peptide were pooled, and lyophilised. The dry weight of the purified peptide was measured to 0.1 μg accuracy using a Sartorius SE2 Ultra Micro Balance and stored at −80°C.

### WaterLOGSY NMR Peptide-Binding Experiments

NMR spectra were collected on a Bruker Avance III 800 MHz spectrometer equipped with a TCI CryoProbe (Bruker) at 298 K in 5 mm glass tubes. Lyophilized aSyn and 4554W were reconstituted in NMR buffer (10 mM sodium phosphate pH 7.0, 100 mM KF, 0.05% NaN_3_) with a final concentration of 5% D_2_O (v/v) to a final concentration of 50 μM and 1 mM, respectively. Each experiment required multiple samples: 1 mM 4554W alone, 50 μM aSyn alone, 50 μM aSyn + 1 mM 4554W. 1D proton and WaterLOGSY spectra were collected on each sample every day for 6 days. Spectra were also obtained using aged peptide (following incubation with a flea at 37°C for 6 days) and aSyn fibrils (formed by incubation with a flea at 37°C for 7 days). The 1D proton spectra were collected with 128 scans, 20 dummy scans, an acquisition time of 0.734 s, and a sweep width of 13.9456 ppm. WaterLOGSY spectra were collected with 256 scans, 16 dummy scans, an acquisition time of 0.729 s, and a sweep width of 14.0396 ppm. 1D and WaterLOGSY spectra were compared in TopSpin 3.5 pl 7 (Bruker) with the control spectra to check for signs of binding. The integral volume for peaks corresponding to the aromatic region (6.5–9 ppm) and aliphatic region (0.5–4.5 ppm) were calculated in Origin and added together to give the peak area per spectra avoiding the water peak. Peak volumes were calculated for spectra obtained for 4554W alone and 4554W plus aSyn (following subtraction of aSyn alone). The difference between the total integral volumes for 4554W and 4554W plus aSyn were calculated and plotted.

### Natural Abundance ^1^H-^13^C Heteronuclear Single Quantum Coherence Spectroscopy (HSQC) of Peptides

4554W was dissolved in D_2_O at 1 mM. 1D proton and ^1^H-^13^C HSQC spectra were collected freshly prepared and following incubation at 37°C with agitation for 6 days. 1D proton spectra were collected with 128 scans, 100 dummy scans, an acquisition time of 0.557 s, and a sweep width of 9.1877 ppm. ^1^H-^13^C HSQC spectra were collected with 16 scans, 16 dummy scans, an acquisition time of 0.100 s (^1^H) and 0.014s (^13^C), and a sweep width of 10.0122 ppm (^1^H) and 65.0005 ppm (^13^C). Spectra were processed in TopSpin and analyzed in CCPN Analysis.

### Transmission Electron Microscopy

Hundred micrometer samples of aSyn were incubated in PBS pH 7.4 alone, or in the presence of equimolar 4554W at 37°C, with agitation with a flea for 1 week. 5μL of each sample was mounted onto a carbon-coated copper grid for 2 min. Excess was blotted away, and grids stained with 4% uranyl acetate for 30 s. Images were collected on a 120 kV Tecnai G2 Spirit BioTWIN electron microscope (FEI) with a SIS Megaview III camera. Image and fibril length analysis was carried out using ImageJ and OriginLab, with the scale bar on each image used as the length reference. Four images were used for each analysis with number of measurements included in each analysis shown in Supplementary Table 2.

### Mass Spectrometry Peptide Analysis

Fresh and aged 4554W samples were diluted to a concentration of 1 pmol/μL in 50% acetonitrile, 0.1% formic acid and infused at 5 μL/min onto the electrospray ionization (ESI) source of an Orbitrap Fusion. Comparison of theoretical and experimental m/z values showed that measurements were accurate to 3 ppm. Asparagine deamidation was probed using the corresponding 1Da mass shift, and the proportion of asparagine deamidated was determined through deconvolution of the isotope (^13^C) cluster peaks. This was further confirmed through higher-energy C-trap dissociation (HCD) MS-MS.

### Thioflavin T Fluorescence

aSyn (WT, A30P, E46K, H50Q, G51D, A53T, and A53E) were prepared in PBS pH 7.4 at a concentration of 100 μM alone and in the presence of 100 μM 4554W. Samples were incubated at 37°C with agitation for up to 1 week. Samples were transferred to a 96-well black-walled, clear bottom plate (Nunc) with 2 μM Thioflavin T and fluorescence measurements taken using λex = 440 nm and λem = 490 nm on a Flexstation 3 microplate reader (Molecular Devices). Each sample was measured in triplicate and the final volume in each well was 50 μL. Data was processed and analyzed using OriginLab. Statistical significance of the differences was assessed using analysis of variance (ANOVA) with the Bonferroni *post hoc* test.

### Addition of 4554W to Pre-formed Fibrils

WT aSyn fibrils were formed by incubating 100 μM aSyn in PBS pH 7.4 at 37°C, with agitation with a flea for 1 week. 4554W was added to equimolar concentration and samples further incubated for 5 days at 37°C with agitation with a flea. Thioflavin T and transmission electron microscopy with image analysis were carried out as described above.

## Results

### 4554W Binds to ‘Partially Aggregated’ aSyn Species and Shows Increased Binding Over Time

We carried out Heteronuclear single quantum coherence spectroscopy (HSQC) experiments using ^15^N labeled aSyn alone and in the presence of increasing concentrations of 4554W, up to a 20:1 peptide:protein ratio. No changes in intensity or chemical peak shifts were observed suggesting little or no binding to “NMR observable” aSyn species (Supplementary Figure 2A). We also observed that when aSyn alone or aSyn plus 4554W was incubated for 6 days the resulting spectra were the same irrespective of the presence or absence of 4554W (Supplementary Figure 2B). This suggests that 4554W does not bind with significant affinity to aSyn species within the NMR visible size range, i.e., oligomers of trimeric or less. It also indicates that the presence of 4554W does not alter the aggregation of these low-n aSyn oligomers, with observed loss of signal upon incubation consistent with aggregation. The peaks that remain correspond to the C-terminal region which is predicted to be outside of the core region of aSyn fibrils (Li et al., 2018) and may remain flexible and therefore NMR accessible upon aggregation. From this data we hypothesized that 4554W could be binding to a larger species of aSyn than cannot be detected via HSQC NMR experiments.

We therefore utilized 1D proton and WaterLOGSY (Water-Ligand Observed via Gradient SpectroscopY) NMR experiments to probe this hypothesis and observe interactions between 4554W and “NMR invisible” aSyn species. These experiments were designed to observe an NMR spectrum of 4554W, with association to aSyn resulting in a reduction in 1D signal as 4554W becomes “NMR invisible.” WaterLOGSY is often employed as a screening method to identify compounds that can interact with proteins or other macromolecules and can be used to probe binding affinities within the micromolar range. The experiment works by transferring magnetization from the bulk solution to the ligand which is then detected as the NMR signal (Dalvit et al., 2000). When some of the ligand is associated with protein, resonances from the bound ligand will be observed with the opposite sign to that of the unbound, identified as reduction in signal.

**FIGURE 1:**
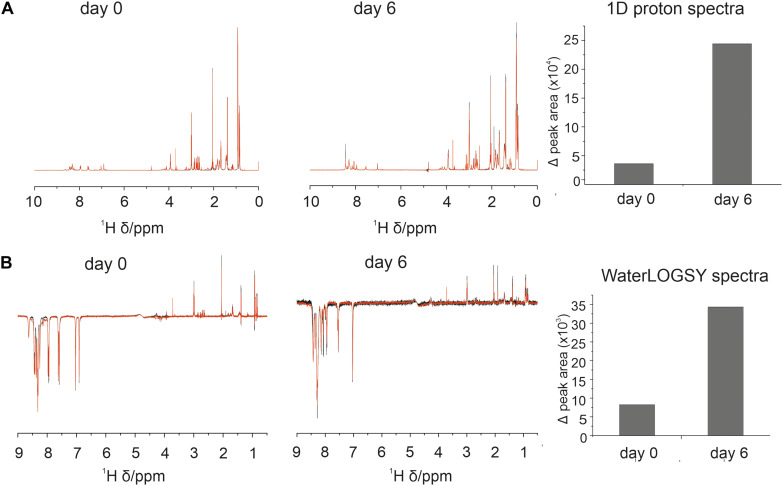
Association between 4554W and aSyn increases over incubation time. **(A)** 1D proton and **(B)** WaterLOGSY NMR spectra collected at day 0 and following incubation for 6 days as shown. The integral volume for peaks corresponding to the aromatic region (6.5–9 ppm) and aliphatic region (0.5–4.5 ppm) were calculated and added together to give the peak area per spectra avoiding the water peak. Peak volumes were calculated for 4554W alone (black) and 4554W + aSyn (red). The difference between the total integral volumes were calculated and plotted (right). A reduction in peak area between 4554W alone and 4554W + aSyn indicates association. The change in peak area is greater at day 6 than day 0 suggesting increased association upon incubation. Difference WaterLOGSY spectra are shown in more detail in Supplementary Figure 3.

1D and WaterLOGSY data indicated some association between 4554W and aSyn at day 0 observed as a difference between the spectrum acquired for 4554W alone and that acquired in the presence of aSyn (Figure 1). Additionally, upon incubation for 6 days the observed difference increased, suggesting enhanced binding over time. This can be seen in the difference WaterLOGSY spectra obtained daily for up to 6 days (Supplementary Figure 3A), with greater peak areas present in the difference spectra at day 6 compared with day 0 (Supplementary Figure 3B). We therefore propose that the peptide “recognizes” and is able to bind to partially aggregated aSyn species and functions to prevent their further aggregation.

### 4554W Becomes Modified Upon Incubation

**FIGURE 2:**
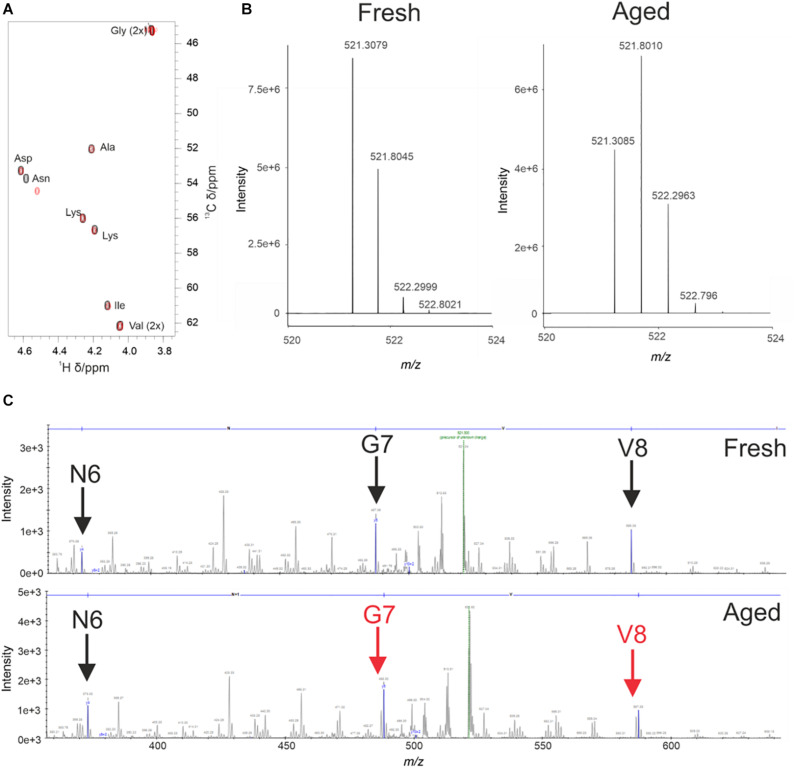
Deamidation of 4554W upon incubation. **(A)** Natural abundance HSQC for fresh (black) and aged (red) 4554W shows a shift in Asn residue. **(B)** Deamidation results in a mass shift of 1Da. Direct infusion mass spectrometry of fresh and aged 4554W showed a shift from primary ion with m/z value 521.3079 (fresh), with less abundant peaks at 521.8045 and 522.2999 reflecting ions containing ^13^C. The aged sample shows a different distribution of peaks, with the predominant peak being the middle peak (521.8010). This suggests that partial deamidation has occurred, and the first ^13^C peak of the unmodified 4554W now overlaps with that of the deamidated 4554W. By looking at the ratio in the relative abundances of each peak in the two samples it was possible to determine the proportion of 4554W that had been deamidated to be approximately 50%. **(C)** Higher-energy collisional (HCD) MS-MS used to determine the location of deamidation by generating ion fragments of 4554W. The distribution of peaks showing a dual population (observed as a preceding peak prior to the main peak, red arrows) for the aged 4554W mimics that seen for the whole peptide at sites C-terminal to the modification site. The lack of preceding peaks in fresh 4554W (black arrows), and loss of the preceding peak corresponding to N6 in the aged 4554W sample indicates that this is the site responsible for the dual population with a difference in mass of 1Da.

During the analysis of WaterLOGSY and 1D data we observed changes in the 4554W spectrum over time (Supplementary Figure 4). To probe this change further ^1^H-^13^C natural abundance HSQC experiments of 4554W alone were performed. The spectra revealed clear shifts in the position of the Cα and Cβ peaks corresponding to the Asn residue (Figure 2A). Asn is prone to deamidation, resulting in a mass shift of 1Da, and is detectable by mass spectrometry (Yang and Zubarev, 2010). Fresh and aged (pre-incubated for 6 days) 4554W, prepared under identical conditions to those used in the NMR experiments, were analzed by MS through direct infusion revealing a primary ion with the m/z value 521.3079 corresponding to the 2 + species (Figure 2B). The fresh 4554W sample gave a predominant peak of 521.3079, with less abundant peaks at 521.8045 and 522.2999 which reflect those ions containing ^13^C. In the aged sample the distribution of these peaks was different, with the predominant peak being the middle peak (521.8010). This suggests that partial deamidation of the sample had occurred, and the first ^13^C peak of the unmodified peptide superimposed with that of the deamidated peptide (observed as + 0.5Da which corresponds to + 1Da in the + 1 species). By observing the ratio of each peak’s relative abundance in the two samples it was possible to determine the proportion of 4554W that had been deamidated to be approximately 50%. Using higher-energy collisional (HCD) MS-MS (Olsen et al., 2007) the location of this deamidation was determined. Unlike in Figure 2B in which m/z values of the whole peptide were recorded, using HCD MS-MS ion fragments of the peptide were generated. As can be seen in Figure 2C, the distribution of peaks mimics that seen for the whole peptide until Asn is removed (Figure 2C). The loss of the preceding peak indicates that the Asn residue is responsible for the dual population (with a difference in mass of 1Da). This corresponds to the mass change induced by deamidation and confirms that Asn 6 in the 10-residue peptide is the site of deamidation. This data highlights that partial deamidation of Asn is taking place within a similar timeframe to the aggregation of aSyn *in vitro* and may be modulating the affinity of interaction between aSyn aggregates and 4554W. The data suggests that greatest binding is achieved between modified peptide and partially aggregated aSyn.

We collected WaterLOGSY NMR spectra using freshly prepared 4554W and following aging to induce deamidation in the presence of freshly prepared aSyn (presumed to be predominantly monomeric) and using aSyn fibrils. Assessment of the difference between 4554W alone and aSyn under these different conditions (Supplementary Figure 5) confirms that binding is enhanced following aging of the peptide, and that greatest binding is observed between fibrillar aSyn and aged 4554W.

### 4554W Prevents Aggregation of PD-Associated Mutants

**FIGURE 3:**
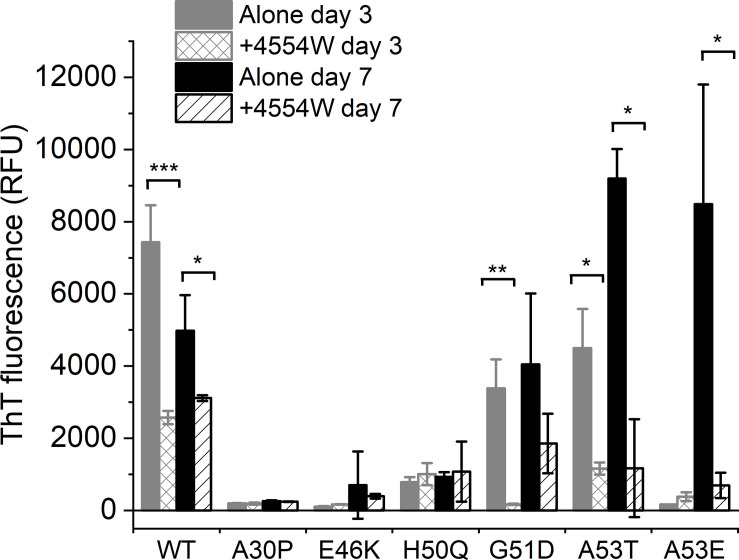
Thioflavin T fluorescence for aSyn variants alone and incubated in the presence of 4554W. WT, G51D, A53T, and A53E show significant reduction in ThT fluorescence upon incubation in the presence of 4554W. Data is shown as mean ± SD for triplicate readings following incubation for 3 and 7 days. Control 4554W alone samples do not show any ThT fluorescence (data not shown). *p < 0.05, **p < 0.01, ***p < 0.001.

The initial peptide screen was designed to target the region containing residues 45–54 of aSyn. This region contains all but one (A30P) of the currently identified PD-associated aSyn mutants (Meade et al., 2019). Here the ability of 4554W to prevent fibrillation of mutant aSyn variants *in vitro* was explored. Thioflavin T (ThT) was used to assess fibril formation of aSyn variants in the absence and presence of equimolar concentrations of 4554W. A significant reduction in ThT fluorescence was observed in the presence of 4554W at day 3 for WT (*p* = 0.0003), G51D (*p* = 0.0023) and A53T (*p* = 0.01082) (Figure 3). A53E took longer to aggregate showing a significant reduction in ThT fluorescence in the presence of 4554W by day 7 (*p* = 0.0216). In contrast A30P, E46K, and H50Q had low ThT fluorescence after 7 days incubation and showed no significant reduction in the presence of 4554W (Figure 3).

**FIGURE 4:**
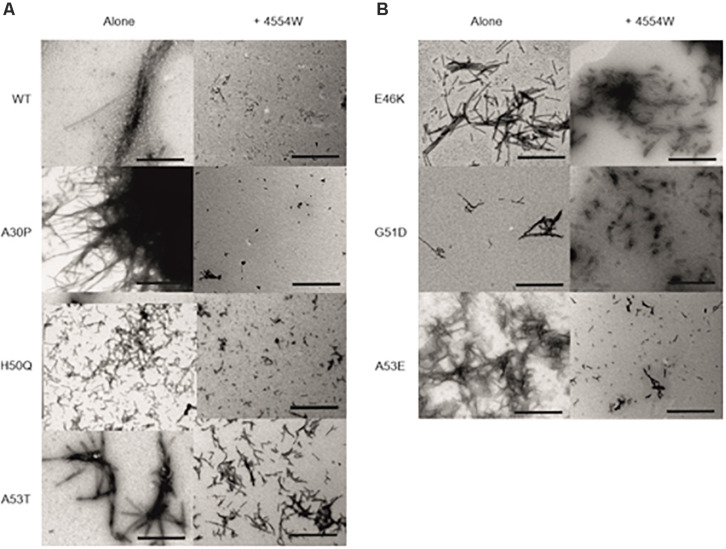
Transmission electron microscopy images for aSyn mutant proteins incubated alone and in the presence of 4554W. **(A)** WT, A30P, H50Q, and A53T show significant reduction in fibril length upon incubation with 4554W. **(B)** E46K, G51D, and A53E do not show significantly altered length in the presence of 4554W. Scale bar is 1μm.

**FIGURE 5:**
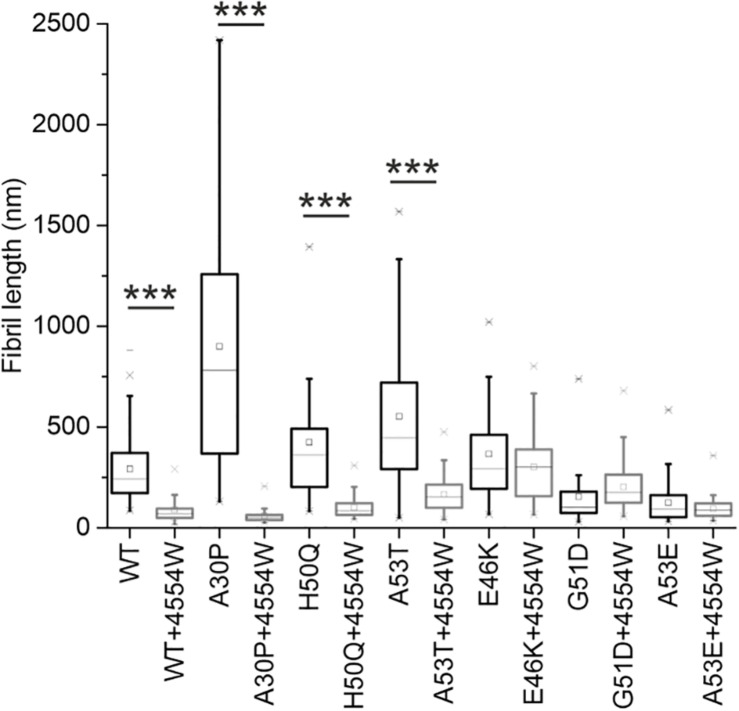
Fibril length analysis for PD-associated aSyn mutants incubated alone and in the presence of 4554W. Lengths of fibrils were measured from TEM images as shown in Figure 4 in ImageJ using the scale bar on each image as the length reference. Four images were analyzed for each condition. Mean length of aggregates observed is shown as a horizontal line with the median shown as a small square. Upper and lower limits of the boxes indicate the 25th and 75th percentiles of the data, with “whiskers” indicating the 5th and 95th percentile range of the data. A30P, H50Q, and A53T show statistically significant (***p ≤ 0.0001) reduction in fibril length in the presence of 4554W. The number of length measurements taken for each condition and *p*-values are shown in Supplementary Table 2 with proportion of each length fibrils shown in Supplementary Figure 6.

TEM analysis was used to observe changes in morphology of fibrils formed following incubation for 1 week *in vitro*. WT, A30P, H50Q, and A53T mutant aSyn variants form long fibrils when incubated alone (Figure 4A). When incubated in the presence of 4554W shorter fibrils/aggregate species were observed, with length analysis confirming most species < 150 nm in length (Supplementary Figure 6), and mean lengths significantly reduced compared to the aSyn variants alone (Figure 5 and Supplementary Table 2). In contrast, addition of 4554W had little effect on the length of fibrils formed from E46K, G51D, or A53E mutants (Figure 4B). G51D and A53E alone produced short fibrils in comparison to WT and other mutant aSyn variants with approximately 50% of fibrils measured < 100 nm.

### 4554W Disaggregates Pre-formed aSyn Fibrils

**FIGURE 6:**
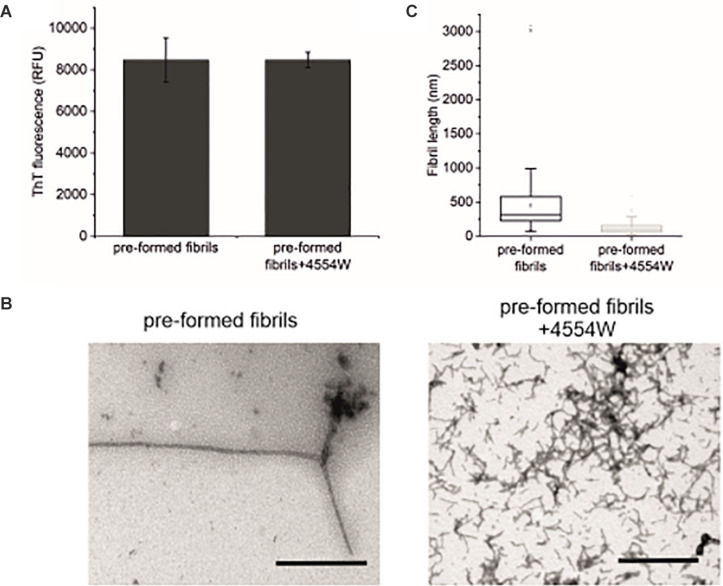
Incubation of pre-formed aSyn fibrils with 4554W. **(A)** No difference in ThT fluorescence was observed following incubation of pre-formed aSyn fibrils with 4554W for 5 days. **(B)** TEM images show short fibrils upon incubation of pre-formed fibrils with 4554W. Scale bar 1 μM. **(C)** Lengths of fibrils measured from 4 images using the scale bar as the length reference show a significant reduction in mean length compared to fibrils incubated alone. Mean length of aggregates observed is shown as a horizontal line with the median shown as a small square. Upper and lower limits of the boxes indicate the 25th and 75th percentiles of the data, with “whiskers” indicating the 5th and 95th percentile range of the data. The number of length measurements and *p*-values are shown in Supplementary Table 2 with proportion of each length fibrils shown in Supplementary Figure 6.

Further to data showing 4554W preferentially associates with aggregated aSyn species (Supplementary Figure 5), the ability of 4554W to disaggregate pre-formed fibrils was probed. Incubation of 4554W with pre-formed WT aSyn fibrils for 5 days showed no alteration in ThT fluorescence (Figure 6A). However, TEM analysis showed a significant reduction in fibril length (Figures 6B,C and Supplementary Figure 6) resulting in fibrils of similar size to those produced when non-aggregated aSyn is incubated in the presence of 4554W (as shown in Figures 4A, 5). This indicated that 4554W can disaggregate pre-formed aSyn fibrils. Taken together work presented here showed that the presence of 4554W resulted in shortened fibrils independent of the starting aSyn species, consistent with the peptide acting to both prevent further aggregation and alter pre-formed aggregates.

## Discussion

4554W was previously shown to inhibit fibrillation and reduce cytotoxicity of aSyn showing potential biological and therapeutic benefit for 4554W (Cheruvara et al., 2015). Here we showed that 4554W can bind to partially or fully aggregated species of aSyn preventing further fibrillation and resulting in shorter fibrils. In contrast no association is observed with monomeric aSyn. We report that maximal binding was achieved when 50% of 4554W had undergone deamidation of the Asn 6 residue. Deamidation can affect the structural integrity of proteins and peptides through the introduction of a negative charge which may affect the ability of 4554W to interact with aSyn and alter self-association interactions. Given that deamidation occurred under *in vitro* conditions that promoted aSyn aggregation we believe that deamidation of the peptide would also occur *in vivo* if 4554W was to be utilized in the future as a therapeutic option. However, an alternative future strategy could involve synthesizing deamidated 4554W which may show improved association with aSyn.

Many avenues targeting aSyn aggregation are under investigation as disease-modifying therapeutic approaches. The small molecule inhibitor Fasudil has been shown to prevent aggregation of aSyn by binding to the C-terminal region identified via specific NMR chemical shifts (Tatenhorst et al., 2016). In this study we report a lack of chemical shift changes following addition of 4,454 W to aSyn showing 4554W doesn’t associate with monomeric asyn. The flavonoid epigallocatechin gallate and phenol curcumin have shown potential for inhibiting several aggregation-prone proteins (Ehrnhoefer et al., 2008; Ahmad et al., 2017). They also act via direct association to prevent the structural re-arrangement required for fibril formation (Meng et al., 2009; Takahashi et al., 2015). Other avenues being explored include the natural antimicrobial, squalamine which has shown promise as an inhibitor by competing with aSyn in binding lipid membranes, specifically inhibiting aggregation initiation and in turn reducing aSyn toxicity in cell and animal models of Parkinson’s disease (Perni et al., 2017).

4554W is observed to reduce fibril formation assessed by ThT or TEM against all known PD-associated mutants except E46K. aSyn mutants that result in shorter fibrils when incubated in the presence of 4554W (A30P, H50Q, and A53T) have been previously shown to enhance the formation of oligomers (Marvian et al., 2019). In contrast mutants that do not show an effect on fibril length have been shown to have no effect (E46K) or an inhibitory effect (G51D and A53E) on oligomer formation. Morphological and structural similarities have also been observed between WT, A30P, and A53T fibrils (Ruggeri et al., 2020). Recent studies have shown that small molecules SynuClean-D and ZPD-2 can reduce the aggregation of WT, H50Q, and A30P (Pujols et al., 2018; Peña-Díaz et al., 2019). These mutants affect aSyn oligomerization, therefore taken together data presented in this study and previous data suggest that 4554W could be acting during the oligomerization step targeting a common assembly pathway in WT, A30P, H50Q, and A53T to modulate fibrillation.

G51D is associated with earlier age of onset, and a slower rate of aggregation, therefore likely persisting in the oligomeric state for longer than WT aSyn (Fares et al., 2014). This is consistent with very few isolated patches of fibrils observed in our analysis. In the presence of 4554W fibrils were more prevalent suggesting that the presence of 4554W may influence G51D aSyn to follow an alternative aggregation pathway that favors the formation of amyloid fibrils, over that of oligomeric species. Therapeutically this may be beneficial as fibrils may represent a benign endpoint. ThT fluorescence data for G51D suggests that oligomers that form in the absence of 4554W have high ThT fluorescence whereas fibrils produced in the presence of 4554W have lower ThT fluorescence. A53E is the most recently identified PD-linked point mutation in aSyn (Pasanen et al., 2014). It is thus the least characterized with little known about its rate of aggregation or fibril morphology. Here, we show that 4554W reduces ThT fluorescence of A53E fibrils but has little effect on fibril length assessed by TEM.

Interestingly, fibrils that show low ThT fluorescence with no significant alteration in the presence of 4554W (A30P and H50Q) show large fibrils with significant reduction by TEM. Whereas, in contrast G51D and A53E show significant reduction in ThT fluorescence with no show significant alteration in size by TEM. It is known that aSyn mutants likely aggregate by different pathways therefore it could be expected that 4554W may act on all mutants in different ways either altering fibril morphology or amount of fibrils formed. We report that mutants that do not show an effect on ThT fluorescence in the presence of 4554W (A30P, E46K, H50Q) had low ThT fluorescence in the absence of 4554W, in comparison to those mutants that showed an effect. The low ThT for these aSyn mutants alone may explain why a reduction in ThT fluorescence in the presence of 4554W was not observed. We also report that mutants that do not show a reduction in fibril length (E46K, G51D, A53E) were smaller in the absence of 4554W, in comparison to those mutants that showed an effect. This suggests that 4554W is unable to reduce the length of mutants that already produce short fibrils. We therefore hypothesize that 4554W acts to reduce the length of long aSyn fibrils and to reduce ThT fluorescence of aSyn species that have high fluorescence.

We also report that addition of 4554W to pre-formed fibrils does not show a reduction in ThT fluorescence, whereas a significant reduction in length was observed by TEM. We hypothesize for pre-formed fibrils that the amount of fibrils remains the same, whereas the morphology and length of the fibrils is altered. Taken together this study highlights the need to investigate multiple aspects of aggregation to generate a holistic view of effect of addition of a potential aggregation inhibitor.

Data presented here is consistent with 4554W associating with partially aggregated aSyn species. This work highlights a key area of research that is hampered by a lack of reliable, readily available techniques available to study interactions with partially aggregated or oligomeric species directly. This barrier is largely due to the transient nature of these aggregation intermediates and the equilibrium that exists between monomeric, partially aggregated and fully formed fibrils. Any attempt to isolate aggregation intermediates to study their interactions causes disruption to this equilibrium and could result in alteration to the species present and an inaccurate representation of what is happening in the native heterogeneous system. Future experiments to probe association of 4554W with oligomeric aSyn species could include ion mobility mass spectrometry as utilized for a range of aggregating proteins (Woods et al., 2013; Young et al., 2015), including aSyn (Liu et al., 2015). Size exclusion chromatography enables isolation of oligomeric species (Daturpalli et al., 2013; Schonhoft et al., 2017) and if coupled with small angle X-ray scattering can provide structural information (Rekas et al., 2010). Employing fluorescent or other labeling approaches would also enable microscopy-based methods to probe and visualize peptide-oligomer interactions. We acknowledge that while we don’t present evidence for direct binding the data we present confirms lack of binding to monomeric aSyn and shows enhanced association upon incubation of aSyn consistent with our conclusion.

The ability of a therapeutic to act after onset of symptoms in neurodegenerative diseases associated with protein aggregation is an important prerequisite for its use as a disease-modifying therapy. This has been shown previously with anle138b able to modulate oligomer formation and inhibit disease progression in a Parkinson mouse model even when treatment was started after disease onset (Levin et al., 2014). Previously identified small molecules have been shown to have different modes of action affecting different stages of aggregation, e.g., ZPD is more effective at early stages of aggregation (Peña-Díaz et al., 2019), whereas SynuClean-D can target later stages (Pujols et al., 2018). Data presented here suggests that 4554W acts following onset of aggregation and is able to disaggregate pre-formed fibrils highlighting its potential for future therapeutic development. Assessing the effect of 4554W on toxicity associated with mutant aSyn aggregation was beyond the scope of the current work. This represents the next stage in development of 4554W as a future therapeutic to confirm the preliminary data presented here highlighting its potential to alter WT and mutant aSyn aggregation. Taken together with additional studies as outlined above probing peptide-oligomer association, toxicity evaluation is required to enable assessment of the effectiveness of 4554W to reduce aggregation associated pathogenicity when added at advanced stages of aggregation and future therapeutic development.

Overall the data suggests that it may be feasible to target aSyn variants with similar fibril morphological properties (WT, A30P, H50Q, and A53T) by a single therapeutic strategy, instead of requiring individual approaches for each PD-associated mutation. The data also provides additional confirmation that peptide therapeutics may be a feasible option for modulating aSyn aggregation, and associated cytotoxicity. This is especially attractive if they can be targeted to pre/partially-aggregated species overcoming the need to administer them prior to signs of disease onset.

## Data Availability Statement

All datasets presented in this study are included in the article/Supplementary Material.

## Author Contributions

JT carried out all experiments included in the manuscript. RMe synthesized the peptides. RMi carried out sample preparation as part of a summer vacation studentship. JMas and JMad conceived the study and acquired funding. All authors were involved in scientific discussion throughout the project. JT and JMad wrote the initial draft with RMe and JMas was involved in re-drafting. All authors shared the responsibility for contributing to the final version of the manuscript.

## Conflict of Interest

The authors declare that the research was conducted in the absence of any commercial or financial relationships that could be construed as a potential conflict of interest.
