# Supplementary material for: Insights Into Peptide Inhibition of Alpha-Synuclein Aggregation
Source: Front Neurosci. 2020 Oct 15;14:561462. doi: 10.3389/fnins.2020.561462 (PMC7594713; doi:10.3389/fnins.2020.561462)
Supplement: Supplementary file 1 [file Data_Sheet_1.docx]

Supplementary Material

# Supplementary Methods

## Peptide-binding NMR titrations

^15^N-labelled WT aSyn was resuspended in NMR buffer (10mM sodium phosphate pH 7.0, 100mM KF, 0.05% NaN_3_) with a final concentration of 5% D_2_O (v/v) such that the final concentration was 180μM. ^1^H-^15^N HSQC spectra were collected and subsequently 4454W (KDGIVNGVKA) peptide was titrated into the sample and experiments repeated until 20:1 peptide:protein ratio. ^1^H-^15^N HSQCs were collected with 4 scans, 32 dummy scans, an acquisition time of 0.109s (^1^H) and 0.061s (^15^N), and a sweep width of 9.1877ppm (^1^H) and 26.0000ppm (^15^N). Spectra were analysed in CCPN Analysis and compared to the assigned WT aSyn spectrum to check for peak shifts or line-broadening.

## Timecourse HSQCs of peptide and α-synuclein

aSyn was resuspended in NMR buffer to a final concentration of 200µM; one sample was of aSyn alone whilst the other contained an equimolar concentration of 4554W. 1D proton and ^1^H-^15^N HSQC spectra were collected on each sample. The samples were then incubated at 37°C with agitation and further spectra collected on the samples every 24hours for 6 days. 1D proton spectra were collected with 32 scans, 8 dummy scans, 0.557s acquisition time, and a sweep width of 9.1877ppm. ^1^H-1^5^N HSQC spectra were collected with 8 scans, 100 dummy scans, an acquisition time of 0.120s (^1^H) and 0.061s (^15^N), and a sweep width of 8.3080ppm (^1^H) and 26.0000ppm (^15^N). CcpNmr Analysis version 2.4.2 (CCPN) was used for data analysis.

# Supplementary Tables

**Table S1: Forward and reverse primers used to generate Parkinson’s disease-associated aSyn mutants**

| Mutation | Primer Sequences |
| --- | --- |
| A30P | 5'-ctcttttgtctttcctggtgcttctgctacaccct-3' 5'-agggtgtagcagaagcaccaggaaagacaaaagag-3' |
| E46K | 5'-catgcaccactcccttcttggttttggagcc-3' 5'-ggctccaaaaccaagaagggagtggtgcatg-3' |
| H50Q | 5'-ctgttgccacaccctgcaccactccctcc-3'  5'-ggagggagtggtgcagggtgtggcaacag-3' |
| G51D | 5'-ccactgttgccacatcatgcaccactccc-3' 5'-gggagtggtgcatgatgtggcaacagtgg-3' |
| A53T | 5'-gtcttctcagccactgtcgtcacaccatgcaccactc-3' 5'-gagtggtgcatggtgtgacgacagtggctgagaagac-3' |
| A53E | 5'-cttctcagccactgtttccacaccatgcaccac-3'  5'-gtggtgcatggtgtggaaacagtggctgagaag-3' |

**Table S2: Numbers of length measurements taken for each condition and presented in Figure 4 and p-values generated by ANOVA between fibril lengths from TEM analysis of fibrils formed in the absence and presence of 4554W. Data is also shown for pre-formed WT fibrils incubated for a further 5 days in the presence of 4554W.**

| **Protein** | **Protein Alone** | **Plus 4554W** | **P value – Protein alone v Protein+4554W** |
| --- | --- | --- | --- |
| WT | 114 | 281 | 6.25273E^-28^ |
| A30P | 39 | 38 | 2.4408E^-18^ |
| H50Q | 46 | 72 | 4.69084E^-21^ |
| A53T | 62 | 68 | 1.16316E^-18^ |
| E46K | 63 | 26 | 0.34879 |
| G51D | 41 | 73 | 0.16097 |
| A53E  WT pre-formed fibrils | 75  114 | 42  274 | 0.18749  2.65546E^-26^ |

# Supplementary Figures


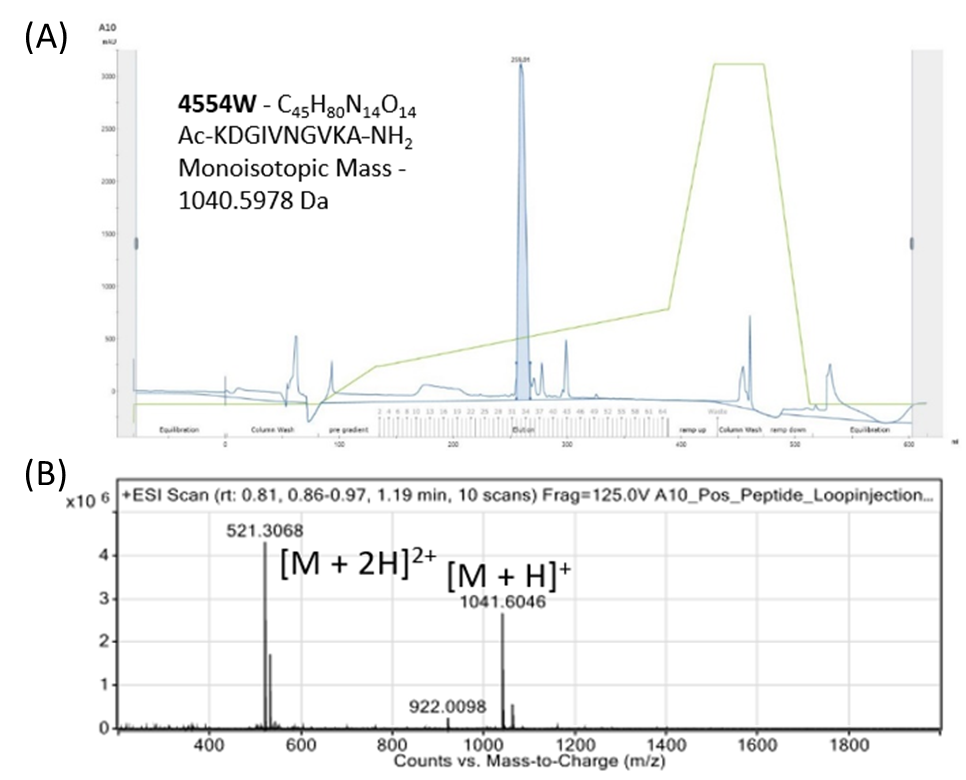


**Figure S1: Production and purification of the 4554W peptide. (A)** HPLC purification trace measured at 215 nm for 4554W. **(B)** Mass spectrometry profile measured by time of flight spectroscopy confirming 4554W mass to be 1040.6 Da, as expected.


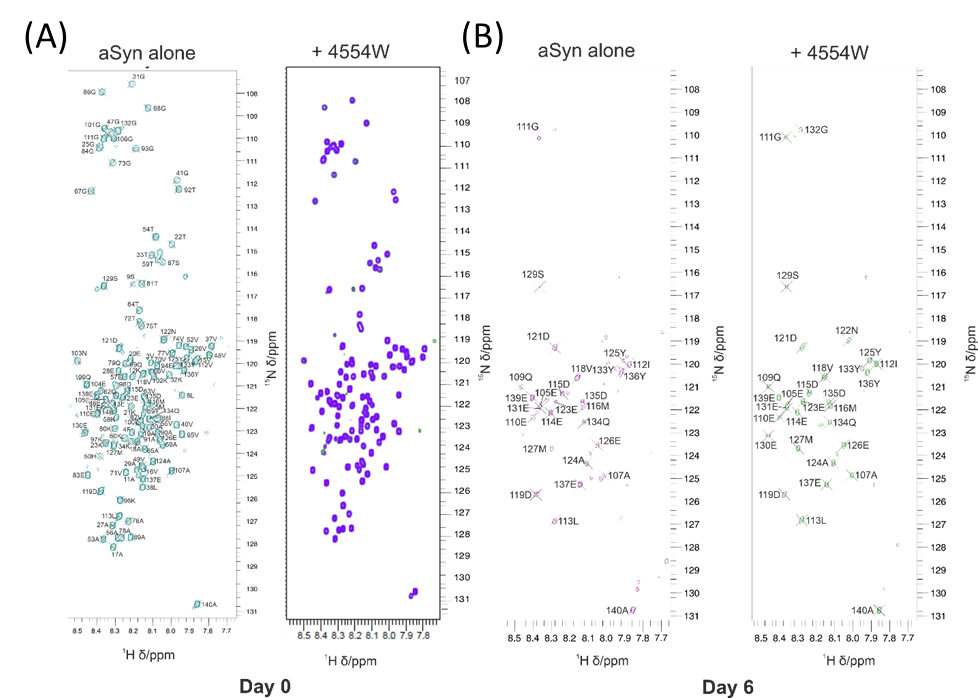


**Figure S2: HSQC NMR spectra of WT aSyn with and without 4554W before (day 0) and after incubation (day 6). (A)** 4554W was added to aSyn up to 20:1 peptide:protein ratio with no observable loss in peak intensity or chemical shift. **(B)** Incubation of aSyn for 6 days at 37°C results in a significant loss of observed peaks with and without 4554W peptide. Those peaks that remain are exclusively located with the C-terminal domain of aSyn, which are highly dynamic and likely have relaxation properties independent of the rest of the molecule.

**
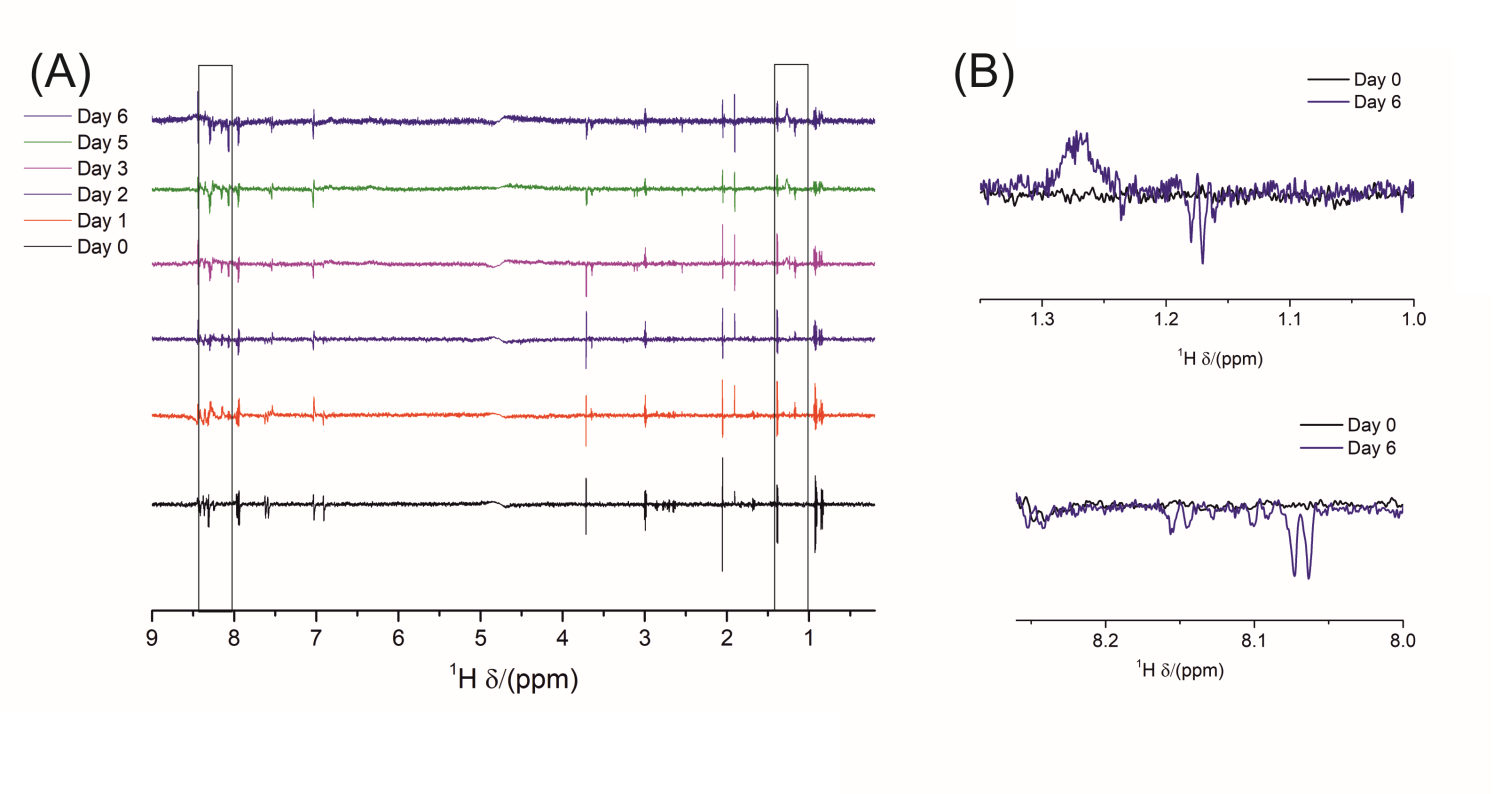
**

**Figure S3: WaterLOGSY NMR spectra shown as difference between 4554W alone and 4554W plus aSyn. (A)** Spectra collected daily show an increase in peak area over time as shown in Figure 1B. **(B)** Key areas showing peaks at day 6 that are absent at day 0 are shown in more detail.


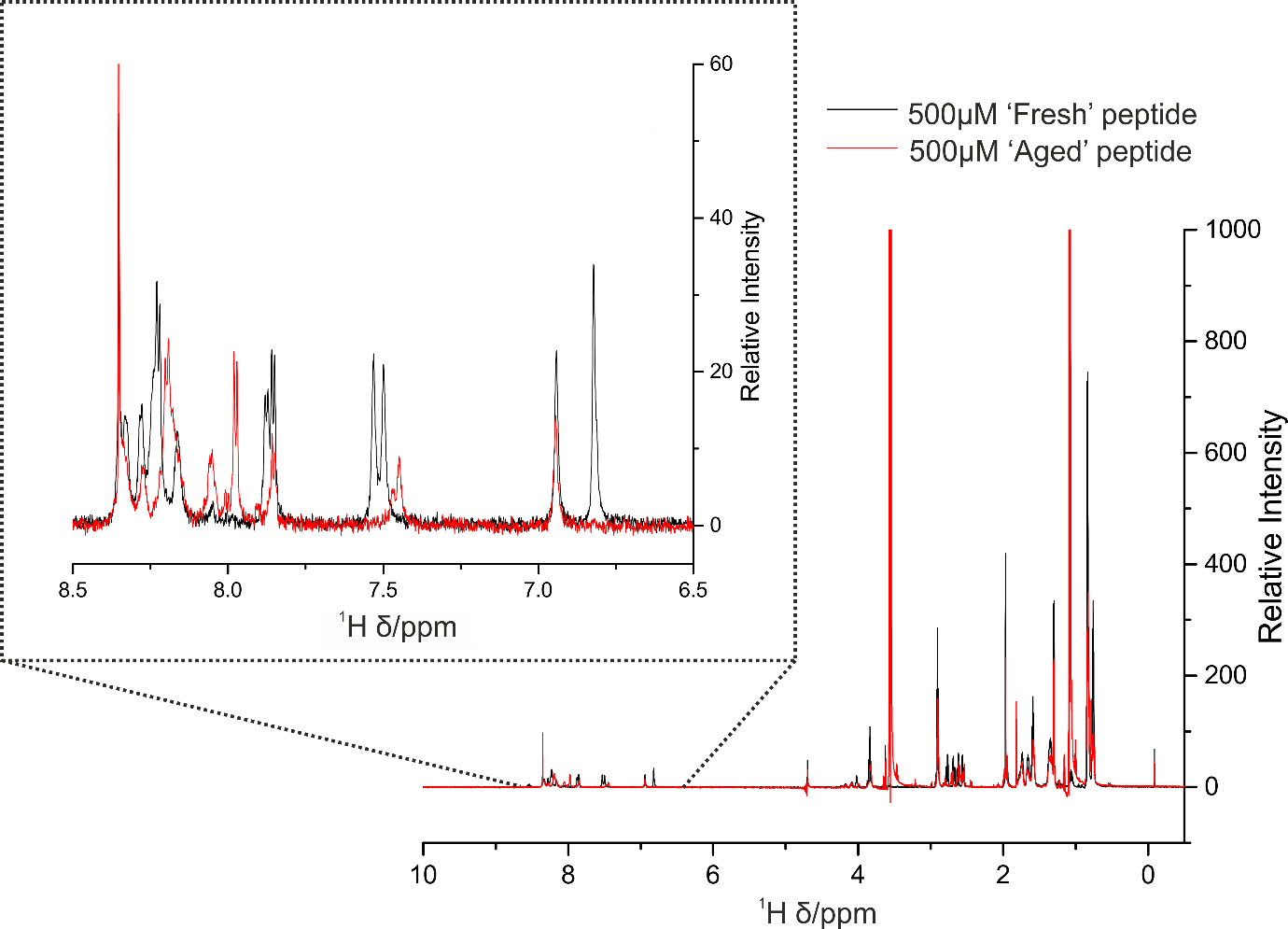


Figure S4: 1D proton NMR spectra of fresh (black) and aged (red) 4554W.

Figure S5: WaterLOGSY NMR spectra shown as difference between 4554W alone and 4554W plus aSyn. Greater difference in the presence of Aged 4554W indicates enhanced interaction with aSyn in both monomeric and fibrillar forms. The greatest interaction is observed between fibrillar aSyn and aged 4554W.

*
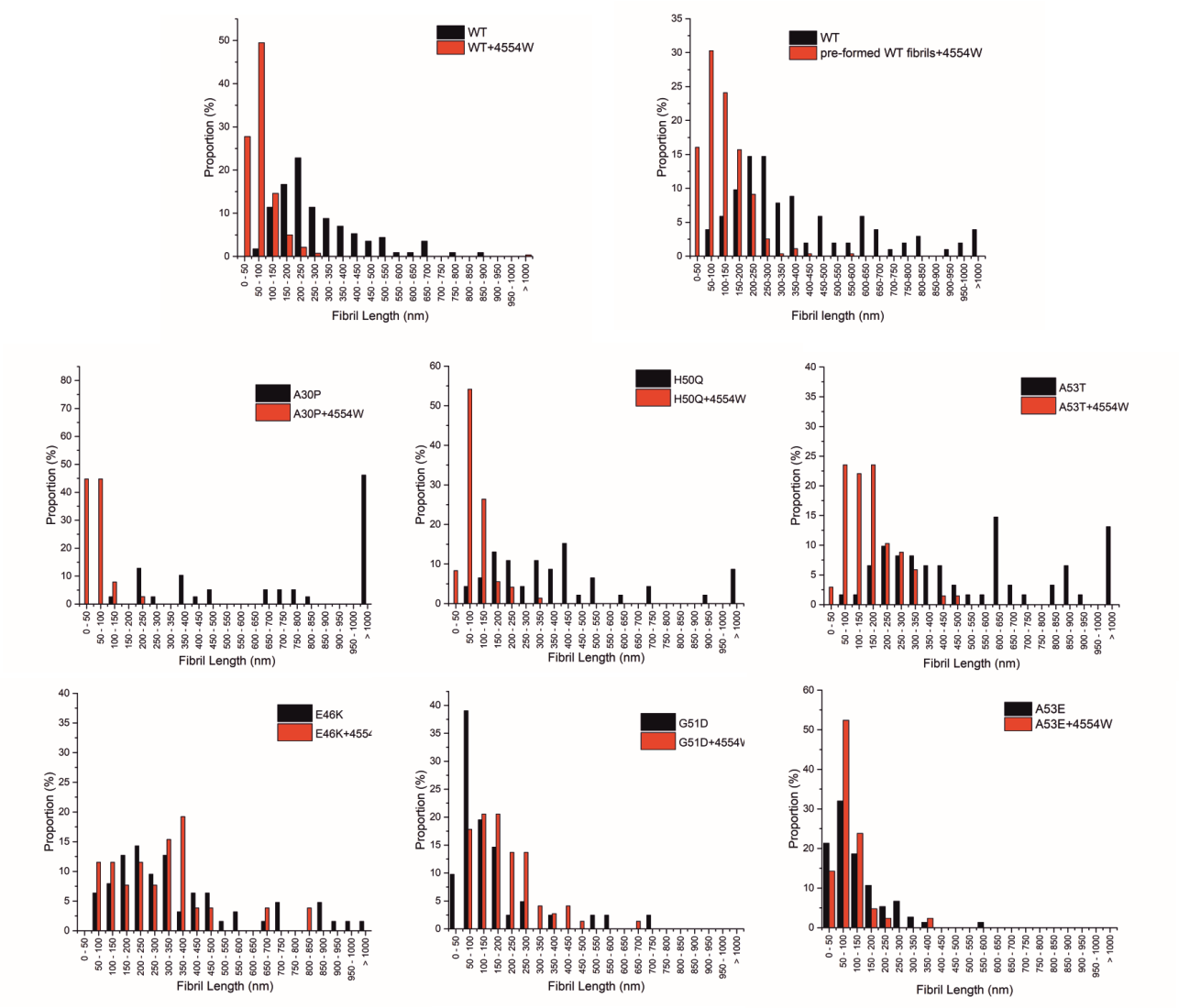
*

**Figure S6: TEM length profiles** **showing proportion of aggregates in each length range measured for PD-associated aSyn mutant proteins alone (black) and in the presence of equimolar 4554W (red). Data is also shown for pre-formed WT fibrils incubated in the presence of 4554W.** Number of fibrils analysed for each condition can be found in Table S2.
